# The parasites of my rival are my friends

**DOI:** 10.3389/fmicb.2023.1135252

**Published:** 2023-05-31

**Authors:** Sigal Orlansky, Frida Ben-Ami

**Affiliations:** School of Zoology, George S. Wise Faculty of Life Sciences, Tel Aviv University, Tel Aviv, Israel

**Keywords:** *Daphnia magna*, *Daphnia similis*, *Hamiltosporidium tvaerminnensis*, host resistance, *Pasteuria ramosa*

## Abstract

The competitive exclusion principle asserts that two species cannot stably coexist in the same habitat. However, the presence of a parasite can facilitate temporary coexistence between two host species occupying the same habitat. Studies of parasite-mediated interspecific competition typically use two host species that are both susceptible to a single parasite species, as it is rare to find a resistant host species that requires a parasite to enable coexistence with a competitively superior susceptible host. We therefore investigated how two host species characterized by different susceptibility profiles affect each other when they coexist in the same habitat, by conducting two long-term mesocosm experiments in the laboratory. We followed populations of *Daphnia similis* coexisting with *Daphnia magna*, in either the presence or absence of the microsporidium *Hamiltosporidium tvaerminnensis* and then the bacterium *Pasteuria ramosa*. We found that in the absence of parasites, *D. magna* competitively excluded *D. similis* within a short period of time. However, in the presence of either parasites, the competitive ability of *D. magna* decreased dramatically. Our results emphasize the importance of parasites in shaping community structure and composition, by allowing coexistence of a resistant host species that would otherwise become extinct.

## Introduction

Parasites are ubiquitous components of ecosystems. They play a key role in determining host species coexistence or exclusion (Holt and Pickering, 1985; Anderson and May, 1986) and thus affect population dynamics, community structure and biodiversity (Hatcher et al., 2006, 2012). Natural host communities are heterogeneous, consisting of individuals that differ in terms of susceptibility, recovery rates, ability to transmit parasites, and the intra community of parasites that they harbor (Hawlena and Ben-Ami, 2015). The dynamics of such communities of hosts and parasites depend on diverse factors, including host–parasite interactions, interactions with other hosts and non-hosts, and environmental factors (Johnson et al., 2015; Louthan et al., 2015). Resources and natural enemies may restrict the existence and distribution of species. The species which has an advantage at the lowest resource level or highest natural enemy pressure will drive all other species extinct (Tilman, 1982; Holt et al., 1994). Two species can coexist provided they differ in the way they affect and are affected by resources and natural enemies. In other word, host coexistence (along with the parasite) requires an appropriate balance among the advantages of each member of the community (Bowers et al., 1994).

In a diversified community, interactions of certain host species with other host species can influence infection prevalence in the host populations (Rudolf and Antonovics, 2005; Keesing et al., 2006) and consequently affect the parasite populations (Ostfeld and Keesing, 2000). Parasites can be instrumental in mediating interspecific competition between host species (Price et al., 1988; Hudson and Greenman, 1998; Hatcher et al., 2006). Their influence may be direct, e.g., by reducing the density or competitive strength of an otherwise competitively superior host in interactions between two host species or between host and non-host species (Park, 1948; Schall, 1992; Norman et al., 1999). The influence of parasites need not be confined to one host species, e.g., *Microphallus* infections altered the behavior of three coexisting species of crayfish, causing all of them to become more vulnerable to predation, albeit the magnitude of the effect of parasitism varied among host species (Reisinger et al., 2015). The influence of parasites may also be indirect (Abrams, 1992), e.g., infections of the dominant herbivorous snail *Littorina littorea* by the digenean trematode *Cryptocotyle lingua* along the northern Atlantic coast of North America reduced its grazing rate and thus indirectly affected the composition of the macroalgal community (Wood et al., 2007). Additionally, parasites can induce enhanced growth of their hosts, i.e., gigantism (Ebert et al., 2004). Larger hosts usually feed faster than smaller ones, and this can influence resource availability, which in turn may influence host resistance and immunity. Furthermore, parasites can induce physiological and behavioral modification to their hosts (host manipulation), and thus influence the overall structure and diversity of communities (Wood et al., 2007).

Although examples of host manipulation by parasites have been widely documented, there are few examples of the effects of a susceptible host population on the composition and structure of a population of resistant host species (hereafter resistant refers to resistance to infection). Resistant species often carry the burden of two types of infection costs: the cost of maintaining defensive mechanisms that incur regardless of whether the individual had been attacked, and the cost of activating a defense mechanism after being attack by a natural enemy (Schmid-Hempel, 2011). In both scenarios, resource allocation to host defense may come at the expense of investment in other life history traits, such as reproduction ability or growth, even in the absence of parasitism (Gwynn et al., 2005). Intensive reproduction could be effective for the maintenance of the population in a competitive environment.

Here we examined the dynamics of water flea populations consisting of a highly resistant species (*Daphnia similis*), in the absence and presence of infected hosts from a susceptible species (*Daphnia magna*). To this end, we used the bacterium *Pasteuria ramosa* and the microsporidium *Hamiltosporidium tvaerminnensis*. The initial driver of the present study was a previous survey of 22 waterbodies in Israel that did not detect any endo- or ecto-parasites in *D. similis*, even though other sympatric crustaceans were found to be infected in those habitats (Goren and Ben-Ami, 2013). In the same survey, as well as in other surveys, *D. magna* was found infected by a variety of parasites (Green, 1974; Ebert, 2005; Goren and Ben-Ami, 2013). Previously we found that the two *Daphnia* species differ in the range and variation of their susceptibilities, and that the parasite produced on average two-fold more spores when growing in *D. magna* clones than in *D. similis* clones (Orlansky and Ben-Ami, 2019). We therefore compared their competitive ability, as this is an integrative trait that includes many other fitness components and has been shown to be a good indicator of *Daphnia* fitness in rock pools (Ebert et al., 2002). We carried out two competition experiments, each with a different parasite species (*P. ramosa* and *H. tvaerminnensis*). These two parasites have different host exploitation and transmission strategies. While *P. ramosa* castrates its host and transmits horizontally upon host death (Ebert et al., 2004), *H. tvaerminnensis* can transmit both horizontally (upon host death) and vertically (from mother to parthenogenetic and sexual offspring) (Vizoso et al., 2005). Furthermore, in the laboratory, *D. similis* is resistant to *P. ramosa*, but it can become infected by *H. tvaerminnensis* at a low rate (S. Orlansky and F. Ben-Ami, unpublished data). The use of these two parasite species can thus provide a broader picture of population dynamics during interspecific competition.

## Materials and methods

To test whether parasites can affect the competitive outcome between populations of resistant vs. susceptible host species, we conducted two experiments using three clones of the resistant host *D. similis* (IL-DSB-6, IL-DSN-3, IL-Sim-A20) and three clones of the susceptible host *D. magna* (BM1, HO2, TY-10). All *D. similis* clones and two of the three *D. magna* clones originated from different geographic and climatic regions in Israel and are known to differ in their infection profile (i.e., genetic basis; Orlansky and Ben-Ami, 2019). HO2 is a widely used laboratory *D. magna* clone that originated from Hungary and is susceptible to infection (Ben-Ami et al., 2008; Ben-Ami and Routtu, 2013; Izhar et al., 2015; Ben-Ami, 2017). In experiment 1, competitions were started by pooling groups of thirty uninfected *D. similis* individuals of two age groups (mature: 12-days-old, and young: 5-days-old, to minimize host age effects; Ben-Ami, 2019) in 24 2 l jars of artificial medium (Klüttgen et al., 1994; Ebert et al., 1998). These 24 jars (populations) were maintained for three weeks to allow them to acclimatize. Six populations included *D. similis* only and thus served as controls. To the remaining 18 populations, we introduced one of three treatments: 12 uninfected *D. magna* individuals, 12 *D. magna* individuals exposed to parasite spores of three *H. tvaerminnensis* isolates (G-3, NZ-2, and FI-OER-3-3), and a mix of six similarly exposed + six uninfected *D. magna* individuals. *H. tvaerminnensis* isolates G-3 and NZ-2 originated from Israel, whereas isolate FI-OER-3-3 originated from Finland (Orlansky and Ben-Ami, 2019). All introduced *D. magna* individuals were 3-weeks-old, and infected *D. magna* were exposed on day five. A separate competition assay (experiment 2) using the abovementioned protocol was conducted with *P. ramosa* clones C1, C19 and C24 (Luijckx et al., 2011). These clones were derived from different *P. ramosa* isolates, and have since been propagated in the laboratory (Izhar and Ben-Ami, 2015; Izhar et al., 2020). Isolates are defined here as parasite samples from infected hosts that may contain multiple genotypes, whereas clones are a single genotype (Luijckx et al., 2011). In both experiments, we used equal proportions of *Daphnia* clones and parasite isolates/clones.

All populations in both experiments were fed with *Scenedesmus* sp. algae cells. To accommodate their growing food demands, from establishment of the populations until the first sample, we increased food levels from 30 × 10^7^ algae cells on the first week, to 45 × 10^7^ algae cells on the second week, and to 55 × 10^7^ algae cells on the third week. Sampling began after 4 weeks, on day 28 of the experiment, and subsequently on a weekly basis, we sampled 6.25% of the animals from each jar using a Folsom Plankton Splitter. After 15 weeks of weekly sampling, we switched to biweekly sampling (sampling weeks 16/18/20/22), as the changes in the populations were small. Sampled animals of each *Daphnia* species from each container were placed in separate jars (to prevent interspecific competition), and adult individuals were separated from young ones to ensure that offspring born after sampling were not included in the sample. We reared the animals for 11 days post-sampling to allow the parasite to develop in case encounter between the parasite and the host had occurred shortly before sampling (Ebert et al., 1996; Hall et al., 2009). Eleven days post-sampling, all animals were counted and scored for infection under a phase contrast microscope (×200–400).

### Statistical analysis

All statistical analysis was done using R version 3.6.3. In both experiments, population densities were treated as longitudinal (repeated measures) data and analyzed using linear mixed models (proc glmer, family Poisson; Bates et al., 2015), with week as a fixed factor, treatment as a categorical variable (*D. similis* kept alone, *D. similis* with 50% or 100% infected *D. magna*, and *D. similis* with uninfected *D. magna*), and jar (population) as a random factor.

## Results

### Experiment 1—*Hamiltosporidium tvaerminnensis*

#### Population dynamics of *Daphnia similis* in the presence of uninfected *Daphnia magna*

**Figure 1:**
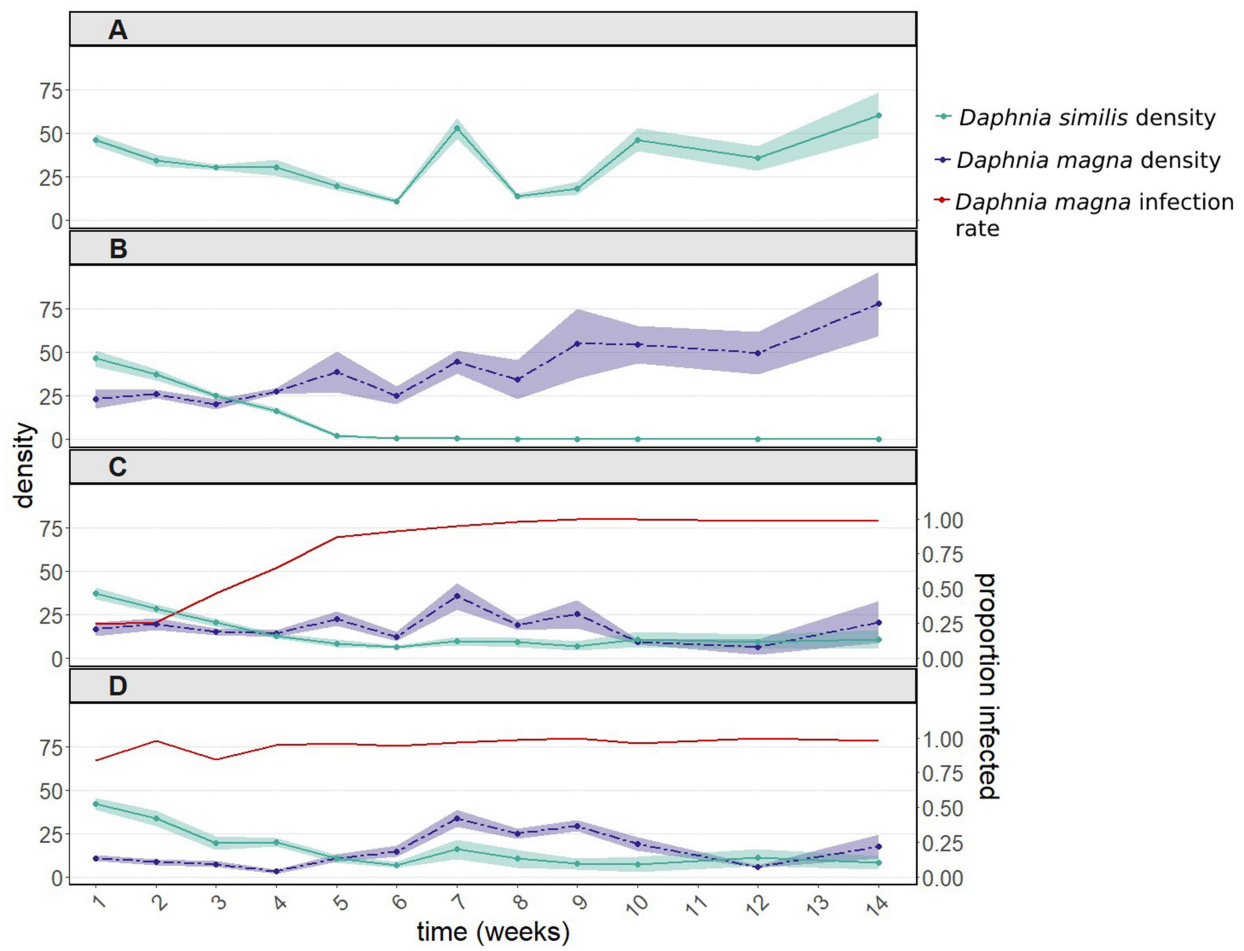
Population dynamics including 95% confidence intervals **(A)** of *Daphnia similis* without interspecific competition, and of both *Daphnia* species **(B)** after introducing uninfected *Daphnia magna*, **(C)** after introducing in equal proportions uninfected and infected *D. magna* (with the microsporidium *Hamiltosporidium tvaerminnensis*), and **(D)** after introducing only infected *D. magna*. Infection rates of *D. magna* by *H. tvaerminnensis* are shown in panels **(C)** and **(D)**. *Daphnia similis* populations remained uninfected throughout the experiment. This experiment was terminated after 14 weekly samplings, when the density of all treated populations (except for the control population – *D. similis* kept alone) had stabilized for several weeks.

In the presence of uninfected *D. magna*, populations of *D. similis* went extinct within 6 weeks (Figure 1B, green line). Such extinction did not occur when *D. similis* populations were kept alone, without parasites and without the competitor (with vs. without uninfected *D. magna*: *z* = 9.211, *p* < 2e-16; Figure 1A, green line). The strong interaction with the sampling week (*z* = −27.113, *p* < 2e-16) is due to the first two weeks, during which there were no significant differences between the two treatments. Thereafter, *D. similis* populations kept alone thrived, while their counterparts under interspecific competition (without parasites) went extinct.

#### Population dynamics of *Daphnia similis* in the presence of *Daphnia magna* infected by *Hamiltosporidium tvaerminnensis*

The population density of *D. similis* was unaffected by the initial infection rates of *D. magna* (50 vs. 100%: *z* = −1.085, *p* = 0.278; interaction with the sampling week: *z* = 1.896, *p* = 0.058; Figures 1C,D, green line). The above nearly significant interaction, when comparing the 50 vs. 100% treatments, is marginal in comparison to the differences in density across weeks (*z* = −16.916, *p* < 2e-16). Furthermore, infection rates of *D. magna* by *H. tvaerminnensis* converged within a few weeks to close to 100% in both 50 and 100% treatments (Figures 1C,D, red line). Therefore, these two treatments were pooled together. The presence of infected *D. magna* prevented the extinction of *D. similis* (pooled infection treatments vs. *D. similis* kept alone: *z* = 1.565, *p* = 0.118), albeit at the end of the experiment, the density of *D. similis* populations with infected *D. magna* was six fold lower than the density of *D. similis* populations kept alone (Figures 1A,C vs. 1D, green line). There were also differences between the density of *D. similis* populations with uninfected vs. pooled infected *D. magna* (*z* = −8.849, *p* < 1e-04), as the former populations went extinct within six weeks (Figures 1B vs. 1C,D, green line). Moreover, the presence of *H. tvaerminnensis* greatly influenced the density of *D. magna*, the treatment populations (Figures 1B vs. 1C,D, purple line).

### Experiment 2—*Pasteuria ramosa*

#### Population dynamics of *Daphnia similis* in the presence of uninfected *Daphnia magna*

**Figure 2:**
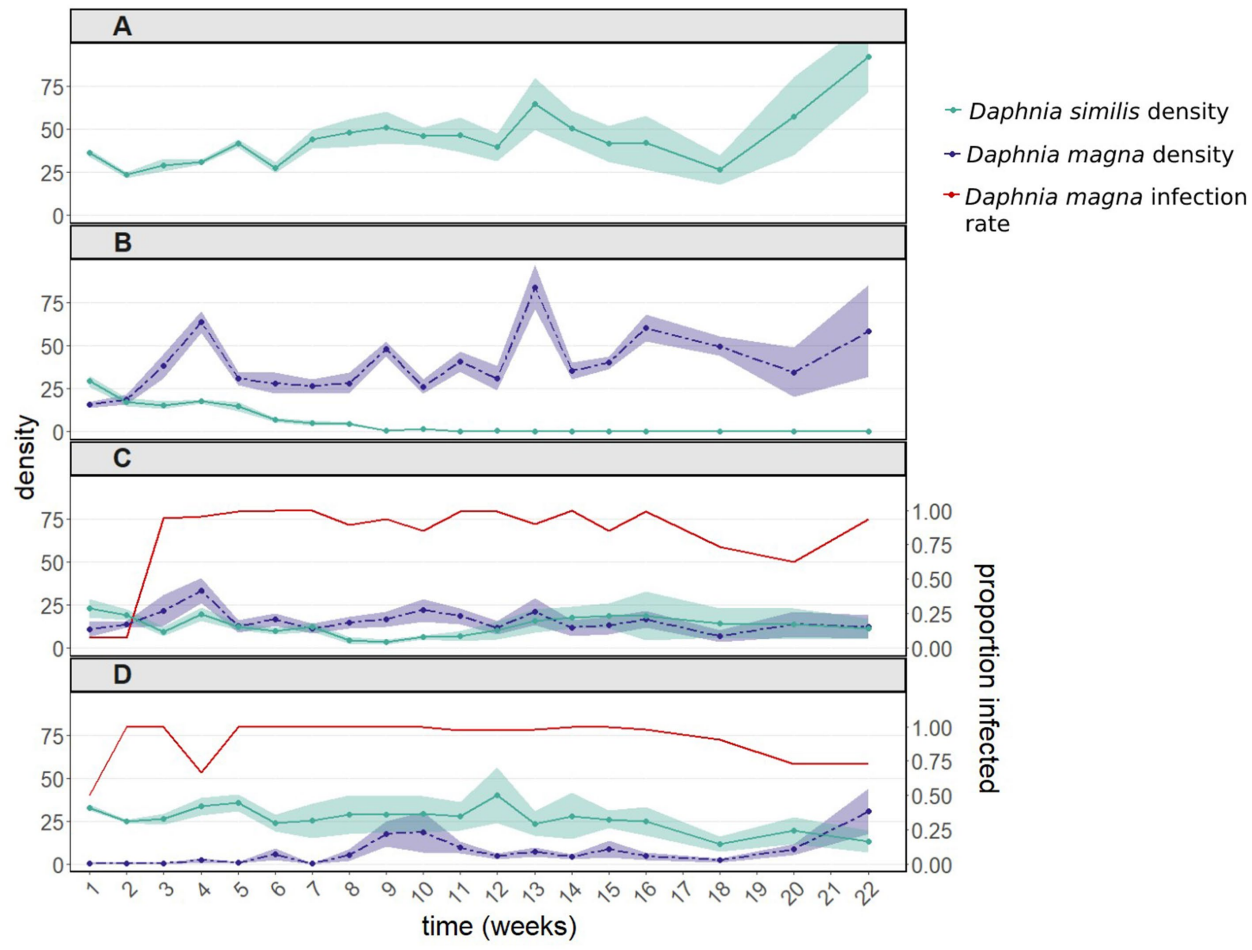
Population dynamics including 95% confidence intervals **(A)** of *Daphnia similis* without interspecific competition, and of both *Daphnia* species **(B)** after introducing uninfected *Daphnia magna*, **(C)** after introducing in equal proportions uninfected and infected *D. magna* (with the bacterium *Pasteuria ramosa*), and **(D)** after introducing only infected *D. magna*. Infection rates of *D. magna* by *P. ramosa* are shown in panels **(C)** and **(D)**. *Daphnia similis* populations remained uninfected throughout the experiment. This experiment was terminated 22 weeks after it had started, when the density of all treated populations (except for the control population – *D. similis* kept alone) had stabilized for several weeks.

As observed in the first experiment, in the presence of uninfected *D. magna*, populations of *D. similis* became extinct within 9–11 weeks (Figure 2B, green line). Such extinction did not occur when *D. similis* populations were kept alone, without parasites and interspecific competition (Figures 2A, green line). Although there was no significant difference between populations with *D. similis* kept alone vs. populations subjected to interspecific competition without parasites (*z* = 1.126, *p* = 0.26), the strong interaction between these two treatments with the sampling week (*z* = −26.124, *p* < 2e-16) indicates that the population dynamics in the presence of interspecific competition without parasites were substantially different from those when *D. similis* populations were kept alone.

#### Population dynamics of *Daphnia similis* in the presence of *Daphnia magna* infected by *Pasteuria ramosa*

In contrast to the first experiment, the population density of *D. similis* was affected by the initial infection rates of *D. magna* (50 vs. 100%: *z* = −2.967, *p* = 0.003; interaction with the sampling week: *z* = 6.020, *p* = 1.8e-09; Figures 2C,D, green line). More precisely, in comparison with *D. similis* populations with 100% infected *D. magna*, which did not differ statistically from *D. similis* populations kept alone (100% vs. *D. similis* kept alone: *z* = −0.034, *p* = 0.973), populations with 50% infected *D. magna* had even lower densities of *D. similis* until week 18 (50% vs. *D. similis* kept alone: *z* = −4.347, *p* = 1.4e-05). Therefore, these two treatments were not pooled together. Despite the differences between the infected *D. magna* treatments, infection rates of *D. magna* by *P. ramosa* converged within a few weeks to close to 100% in both treatments (Figures 2C,D, red line). Nevertheless, both treatments with infected *D. magna* did not result in the extinction of *D. similis* populations (Figures 2C,D, green line). Furthermore, the strong interactions with the sampling week in both treatments (50%: *z* = −6.907, *p* = 5.0e-12; 100%: *z* = −16.993, *p* < 2e-16) indicate that the population dynamics in the presence of both *P. ramosa* and interspecific competition were substantially different from those of *D. similis* populations kept alone (Figures 2A vs. 2C,D, green line). Similar to experiment 1, the presence of *P ramosa* greatly influenced the density of *D. magna*, the treatment populations (Figures 2B vs. 2C,D, purple line).

## Discussion

We found that infections by both *H. tvaerminnensis* and *P. ramosa* can mediate interspecific competition between *D. similis* and *D. magna*. When parasites were absent, *D. magna* competitively excluded *D. similis* within 6–11 weeks. However, the presence of parasites allowed coexistence of both host species. Our results highlight the importance of parasites in shaping host community composition and population dynamics.

The infection rate of the introduced host competitor and the parasite species used to infect the introduced host both influenced the population dynamics of *D. similis*. Whereas the infection rate of introduced *D. magna* infected with *H. tvaerminnensis* did not affect the outcome of interspecific competition, the introduction of 100% *D. magna* infected with *P. ramosa* caused a 2–4 fold increase in the density of *D. similis* during the first half of the experiment, in comparison with introducing 50% infected *D. magna* and 50% uninfected *D. magna*. Thereafter, in the second half of the experiment, the two infection treatments converged to the same level of *D. similis* density, which was fourfold lower than that of *D. similis* populations without *D. magna*. *P. ramosa* is a castrating parasite, hence, the population of infected *D. magna* that was introduced is expected to decrease. Since the population of 100% *D. magna* infected with *P. ramosa* has considerably fewer offspring than the population with 50% infected *D. magna,* resource availability to *D. similis* is expected to increase and can cause its populations to grow in size. However, *P. ramosa* enhances reproduction before castration (fecundity compensation), thereby producing few uninfected offspring (Vale and Little, 2012). Additionally, the parasite causes enhanced growth of its host (gigantism) (Ebert et al., 2004), which usually results in individuals that feed faster than smaller ones. Since the effects of fecundity compensation occur 30–50 days before the effects of gigantism, this may explain why the two infection treatments eventually converged to the same level of *D. similis* density.

An alternative explanation for the differential effects of the infection rate of the introduced host competitor might be related to differences in the susceptibility of *D. similis* to the two parasite species. In comparison with *P. ramosa*, which only infects *D. magna*, *H. tvaerminnensis* can also infect *D. similis*, albeit at a low rate (Orlansky and Ben-Ami, 2019). This microsporidium reduces the fecundity of infected *D. magna* by about 20% (Bieger and Ebert, 2009) as well as enhances reproduction in exposed but uninfected *D. similis* individuals (S. Orlansky and F. Ben-Ami, unpublished data). Although our study did not include a treatment consisting of *D. similis* populations exposed to *H. tvaerminnensis*, it is unlikely that this microsporidium would drive such populations to extinction without the presence of *D. magna*, given its low infectivity and virulence (Orlansky and Ben-Ami, 2019). Additionally, *P ramosa* castrates its host and transmits only upon host death (i.e., semelparous reproductive schedule; Ebert and Weisser, 1997), whereas *H. tvaerminnensis* can also transmit vertically from mother to offspring. In other words, it is in the interest of *H. tvaerminnensis* to keep its host population reproducing. Therefore, possibly due to these effects on the fecundity of both hosts, the introduction of 50% vs. 100% infected *D. magna* did not change the population dynamics of *D. similis*, but still allowed coexistence of both host species.

Interspecific competition among *Daphnia* species (including rotifers as competitors) has been widely studied (Bengtsson, 1986; Östman, 2011; Del Arco et al., 2015; Viaene et al., 2015). Predation, temperature, food conditions and chemical stress have been mostly suggested as drivers of competitive success, albeit competitive exclusion is not always the outcome. Decaestecker et al. (2015) investigated the effects of parasitism and nutrient levels on interspecific competition between *D. magna* and *D. pulex*. Consistent with our study, they found that *D. magna* was competitively dominant to *D. pulex* in the absence of parasites (at both low and high nutrient levels), but the introduction of parasites reversed the competitive hierarchy between these two species. In their study, Decaestecker and coworkers used a solution of three parasite species (the microsporidia *Ordospora colligata* and *Binucleata daphniae*, and the *Daphnia* iridescent virus 1, the causative agent of White Fat Cell Disease). Transmission of these three parasites is strictly horizontal through spores released from the remains of dead, formerly infected hosts (Ebert, 2005), whereas in the present study we used *H. tvaerminnensis*, which also transmits vertically (from mother to offspring) and can thus quickly deplete the reservoir of susceptible individuals. Furthermore, our use of *P. ramosa*, a castrating parasite that strongly reduces host fecundity, provides further support for parasite-mediated interspecific competition.

Competition reduction is an indirect effect of the parasite on the species with which the host interacts (Dunn et al., 2012). Theoretical studies demonstrated that the indirect effects of parasites could exert powerful forces on community composition, facilitating coexistence or promoting exclusion (Holt and Pickering, 1985; Holt et al., 1994). In the presence of a horizontally transmitted parasite such as *P. ramosa*, coexistence with non-host species may be advantageous to a susceptible host, since host–parasite contact rates are reduced (Ebert et al., 2000; Pulkkinen, 2007). However, in the presence of *H. tvaerminnensis*, which transmits both horizontally and vertically, it seems that the reduction of host–parasite contact rates has no advantage, as offspring are born infected. However, the presence of different host species may have an indirect effect of decreasing the intake of spores in crowded conditions.

Our results demonstrate the importance of parasites in shaping community structure and composition. We show that parasites can mediate competition between *Daphnia* species and thus may facilitate coexistence of a relatively resistant host species with an otherwise competitively superior susceptible host species. Given that *D. magna* is the most abundant daphniid in Israel, and yet *D. similis* has been found in many freshwater bodies (Goren and Ben-Ami, 2013), our study shows that parasite-mediated coexistence is possible in this system. A better understanding of systems involving multi-host and non-host species have significant implications for biological invasions and may assist in mitigating the threat from pathogens to endangered species and humans.

## Data availability statement

The raw data supporting the conclusions of this article will be made available by the authors, without undue reservation.

## Author contributions

SO: conceptualization, data curation, methodology, formal analysis, investigation, writing—original draft, review, and editing. FB-A: conceptualization, funding acquisition, methodology, resources, writing—review and editing, and supervision. All authors contributed to the article and approved the submitted version.

## Conflict of interest

The authors declare that the research was conducted in the absence of any commercial or financial relationships that could be construed as a potential conflict of interest.

## Publisher’s note

All claims expressed in this article are solely those of the authors and do not necessarily represent those of their affiliated organizations, or those of the publisher, the editors and the reviewers. Any product that may be evaluated in this article, or claim that may be made by its manufacturer, is not guaranteed or endorsed by the publisher.
